# Understanding the Costs of Surgery: A Bottom-Up Cost Analysis of Both a Hybrid Operating Room and Conventional Operating Room

**DOI:** 10.34172/ijhpm.2020.119

**Published:** 2020-07-27

**Authors:** Sejal Patel, Melanie Lindenberg, Maroeska M. Rovers, Wim H. van Harten, Theo J.M. Ruers, Lieke Poot, Valesca P. Retel, Janneke P.C. Grutters

**Affiliations:** ^1^Department of Operating Rooms, Radboud Institute for Health Sciences, Radboud University Medical Center, Nijmegen, The Netherlands.; ^2^Division of Psychosocial Research and Epidemiology, The Netherlands Cancer InstituteAntoni van Leeuwenhoek, Amsterdam, The Netherlands.; ^3^Department of Health Technology and Services Research, University of Twente, Enschede, The Netherlands.; ^4^Department for Health Evidence, Radboud Institute for Health Sciences, Radboud University Medical Center, Nijmegen, The Netherlands.; ^5^Department of Surgery, The Netherlands Cancer Institute-Antoni van Leeuwenhoek Hospital, Amsterdam, The Netherlands.; ^6^Department of Medical Technology, Isala Hospital, Zwolle, The Netherlands.

**Keywords:** Hybrid Operating Room, Costs, Surgery, Bottom-Up

## Abstract

**Background:** Over the past decade, many hospitals have adopted hybrid operating rooms (ORs). As resources are limited, these ORs have to prove themselves in adding value. Current estimations on standard OR costs show great variety, while cost analyses of hybrid ORs are lacking. Therefore, this study aims to identify the cost drivers of a conventional and hybrid OR and take a first step in evaluating the added value of the hybrid OR.

**Methods:** A comprehensive bottom-up cost analysis was conducted in five Dutch hospitals taking into account: construction, inventory, personnel and overhead costs by means of interviews and hospital specific data. The costs per minute for both ORs were calculated using the utilization rates of the ORs. Cost drivers were identified by sensitivity analyses.

**Results:** The costs per minute for the conventional OR and the hybrid OR were €9.45 (€8.60-€10.23) and €19.88 (€16.10- €23.07), respectively. Total personnel and total inventory costs had most impact on the conventional OR costs. For the hybrid OR the costs were mostly driven by utilization rate, total inventory and construction costs. The results were incorporated in an open access calculation model to enable adjustment of the input parameters to a specific hospital or country setting.

**Conclusion:** This study estimated a cost of €9.45 (€8.60-€10.23) and €19.88 (€16.10-€23.07) for the conventional and hybrid OR, respectively. The main factors influencing the OR costs are: total inventory costs, total construction costs, utilization rate, and total personnel costs. Our analysis can be used as a basis for future research focusing on evaluating value for money of this promising innovative OR. Furthermore, our results can inform surgeons, and decision and policy-makers in hospitals on the adoption and optimal utilization of new (hybrid) ORs.

## Background

**Key Messages**

**Implications for policy makers**

- In an era where sustainable healthcare is an important policy issue, cost drivers and accurate cost estimates of hybrid operating rooms (ORs) are lacking.
- The costs of the hybrid OR were mainly driven by the inventory and construction costs and its utilization rate.
- As the hybrid OR is substantially more expensive (€18.84 vs. €9.45), decision-makers and surgeons should be aware of the increased costs of the use of highly advanced ORs and weigh the extra costs with the health gain.
- Besides providing insight in the costs of the OR, this paper can be used as a basis for future research focusing on evaluating the added value of the hybrid OR.

** Implications for the public**

We performed a comprehensive bottom-up cost-analysis to inform clinicians and policy-makers on the costs and cost drivers of an (hybrid) operating room (OR), as a first step in evaluating the added value of the hybrid OR. As the hybrid OR is substantially more expensive (€18.84 vs. €9.45), decision-makers and surgeons should be aware of the increased costs of the use of highly advanced ORs and weigh the extra costs with the health gain. Besides providing insight in these costs, our analysis can be used as a basis for future research focusing on evaluating value for money of this promising innovative OR.

Over the past decade, many academic and teaching hospitals have adopted a hybrid operating room (OR), and many others are considering it. The compound annual growth rate for the coming 5 years (2019-2023) of the hybrid OR market growth was estimated at 12.5%.^1^

The hybrid OR claims to improve efficiency by means of reducing secondary procedures and improve surgical performance which results in improved clinical outcomes.^2-5^ Hybrid ORs are currently mainly used for cardiovascular surgery,^2-4^ but for neurosurgery^6^ and surgical oncology^7^ interest is increasing. The first observational studies in cardiovascular surgery verified this promising nature by showing a reduced length of stay and reduced operation time.^8,9^ Adoption of such an OR however is a large investment. Since surgical healthcare expenditures already account for a large part of the annual healthcare costs, these innovative ORs have to prove themselves in terms of value for money.^10-12^

In evaluating the added value or cost-effectiveness of the hybrid OR, it is crucial to have insight in its incurred costs. So far, the costs related to the hybrid OR have not been studied. Furthermore, to put suchcosts into perspective, it is important to also gain insight into the incurred costs related to the conventional OR setting. Current estimations on the costs of a conventional OR report a great variety, ranging from $7 to over $100 per minute.^13,14^ The variety can be explained by differences in study design such as the inclusion of different cost categories eg, expensive implants, medical devices, robotics, and site differences as being a (non)teaching hospital, the number of available ORs, the occupancy rate and healthcare system (country-specific). These site characteristics can especially have an influence as OR costs are mainly evaluated top-down. As this method is known to provide little insight into cost drivers and often results in inaccurate cost estimates,^15^ a bottom-up cost analysis is proposed for further research. This method is used to provide more insight into the cost drivers and enable optimization of processes which could result in cost reductions.^15-19^

The aim of this study was to perform a comprehensive bottom-up cost-analysis to inform clinicians and policy-makers on the costs and cost drivers of an (hybrid) OR as a first step in evaluating the added value of the hybrid OR. The results are incorporated in a calculation model to enable usage and adjustment of the input parameters to a specific hospital or country setting (Supplementary file 1).

## Methods

The bottom-up cost-analysis was conducted following the Dutch guideline for costing analyses.^20^ This guideline contains reference prices and formulas to estimate costs related to personnel, equipment, construction, and overhead. The analysis was conducted in five Dutch hospitals, all equipped with both a conventional and hybrid OR. Specific characteristics of the included hospitals are described in Supplementary file 2. Per hospital, we evaluated the following cost categories: Construction costs (2.1), inventory costs including medical devices (2.2), personnel costs (2.3), and overhead (2.4) for a conventional and a hybrid OR. The construction, inventory, and personnel costs were evaluated by interviews and hospital-specific data such as invoices and utilization data. By using hospital-specific invoice data we could perform a bottom-up analysis, instead of using total annual expenses which is often done in a top-down analysis. Supplementary file 2 also reports the positions of the involved experts in these interviews.

 In this study, the conventional OR was defined as an OR where open procedures are performed to evaluate the costs of a basic OR environment. Endoscopic specific devices, to perform laparoscopic and robotic procedures, were thus left out of the analysis. The hybrid OR was defined as an OR in which an imaging technique – at least a fixed C-arm – is installed.

###  Construction Costs

In constructing an (hybrid) OR, each hospital makes specific choices for its design because of, among other things, architectural preferences or limitations, preferences for ventilation systems, country-specific legislation, and budget constraints. To avoid such hospital-specific differences, we estimated total costs for constructing a square meter (m^2^) of an OR based on Dutch key numbers presented by the Dutch advisory board on healthcare housing.^21^ Following these key numbers, constructing a standard m^2^ in a hospital costs €3479 in 2018. For the OR department, these costs should be differentiated with 160%, resulting in a cost of €5.595 per m^2^(C_m2 costs OR department_).

This differentiated cost results in the costs of a m^2^ which does not take into account the different functionalities available within the OR department (corridors, stockrooms, offices, holding and recovery department and the ORs). To estimate the specific costs for a m^2^ of OR per hospital, we identified the total m^2^ of each of these specific functionalities within the OR department based on floor plans (eg, total m^2^of offices). The costs of these m^2^ were calculated using their specific differentiation based on key numbers and expert opinions, such as 140% for holding and recovery, 75% for corridors and offices as described in Table 1. The general m^2^ price of an OR per hospital was calculated by:

**Table 1:** Input Parameters

**Parameter**	**Input Value**	**Input Value Specific Per OR Setting**		**Source**
		**Conventional(Range)**	**Hybrid(Range)**	
General				
Surface of OR (m^2^)		48.5 (40.6-57.0)	85.4 (52.3-106.2)	Hospital data
OR availability per year (min)		122 400	122 400	Hospital data and available hours assumed to be the same for the hybrid OR
Utilization rate of OR (%)		92 (87-96)^a^	43 (14-55)^a,b^	
Construction				
Costs for a general m^2^ in a hospital (€)	3 479			^21^
Differentiation rate over a general square meter in a hospital per category (%):				
OR department	160			^21^ and expert opinion
Holding	140			
Recovery	140			
Corridor and offices	75			
Technical rooms	75			
Sanitary/washing rooms	100			
Annual interest (%)	4.2			^20^
Lifespan (y)	25			^20^
Maintenance (%)	5			^20^
Inventory				
Lifespan (years)	10			^20^
Maintenance for general inventory (%)	5			^20^
Maintenance for medical imaging devices (%)	8			Expert opinion
Personnel				
Costs per hour (€) academic setting			
Medical specialist	117.59			^20,23^
Medical assistant	36.84			
Technician			36.84	
Costs per hour (€) general hospital			
Medical specialist	120.71			^20,23^
Medical assistant	38.06			
Technician			38.06	
Annual loaded working hours				^20^
Medical specialist	2 100			
Medical assistant	1 558			
Technician			1 558	
Overhead				
Calculated over construction and personnel (%)	38			^20^

Abbreviation: OR, operating room. ^a^Based on only 4 hospitals. ^b^Utilization rate of hybrid OR has a slightly different definition than the utilization rate of the conventional OR: annual number of procedures with the use of the C-arm divided by the total annual procedures in the hybrid OR, multiplied with the utilization rate of a conventional OR.


(1) $$\sum C_{M2\;not\;labeled\;as\;OR}=\sum M_{functionality\;i}^{2}*C_{differentiated\;i}$$

(2) $$\frac{\left(\sum M_{OR\;department}^{2}*C_{M2\;\cos ts\;OR\;department}\right)-\sum C_{M2\;not\;labeled\;as\;OR}}{\sum M_{OR\;within\;OR\;department}^{2}}$$

The “C” in this formula refers to costs and the “M^2^” to the square meters. An example of the calculation can be found in Supplementary file 3.

To estimate the construction costs of both ORs, the average m^2^ costs of an OR were multiplied with the mean surface of a conventional OR and a hybrid OR including the control room, based on data from the participating hospitals. Yearly costs of interest and amortization were calculated by using a life span of 25 years and an interest rate of 4.2%. A5% maintenance cost over the construction costs was included.^20^

###  Inventory Costs Including Medical Devices

 For each hospital the inventory for the conventional and hybrid OR was identified, comprising all equipment standing and hanging in the OR such as operating table, operating lights, (computer) screens, chairs, instrument tables, step stools, and closets. To evaluate the actual (negotiated) costs, the equipment and inventory were linked to the actual acquisition costs paid by each hospital based on their recent invoices (including value-added tax [VAT] and discounts). These costs were categorized as follows: general inventory, anesthesia equipment, OR lights, arm pendants, OR table, image routing system, X-ray radiation protection aprons, and fixed C-arm.

All costs made before 2018 were converted to 2018 Euro by using the consumer price index value for the Netherlands.^22^ Per cost category, average costs were calculated to determinethe average inventory cost for each OR. The yearly costs of interest and amortization were calculated using a depreciation period of 10 years, and interest rate of 4.2%.^20^ Yearly maintenance costs of 5% of the average acquisition costs for general inventory and 8% for imaging equipment were included (Table 1). The percentage of mainentance costs for imaging equipment was based on expert opinion and only focusses on costs directly related to the inventory, as no formal estimate is known for any other additional cost such as personnel costs.

###  Personnel Costs

 The personnel costs were based on the number of staff needed for a surgical procedure. We used this approach, and not the actual annual personnel spending of the different hospitals, to overcome the differences between the hospitals.

Per hospital, we identified the composition of the OR team that is available during a surgery in general in the conventional OR and the hybrid OR. The personnel costs per hour were calculated by dividing the total number of effective working hours per year of each function by the total annual loaded salary.^20^ The total annual loaded salaries were retrieved from collective labor agreements for academic and general hospitals.^23,24^ The costs of a medical specialist were obtained from the Dutch guideline.^20^ To account for costs related to irregular working hours, holiday allowance and social security, the salarieswere corrected by a percentage of 39% for general personnel and 35% for higher (medical) personnel. The total personnel costs were calculated by taking the mean of these hospital estimations.

###  Overhead

Overhead expenses, the costs that are not directly attributable to a particular resource but are essential in providing care eg, electricity, water, cleaning service, and administrative tasks, were only calculated over the construction costs and personnel costs to avoid double counting. We used the general percentage for overhead on the direct costs for medical departments of 38%, as recommended by the Dutch guideline.^20^ The expected higher overhead costs for the hybrid OR (eg, larger demand in electricity) are incorporated by calculating the overhead over the average construction costs because the surface of a hybrid OR is larger than the surface of the conventional OR.

###  Analysis

The costs from the bottom-up cost analysis, except the costs for personnel, are expressed in yearly costs. Those are the average costs of the five consulted hospitals. To calculate the total costs per minute, it was needed to combine the average total yearly costs with the average utilization rate. Therefore, the available hours and occupied hours of a conventional OR were identified per hospital for 2018. This resultedin an average utilization rate of the conventional OR. As cross-functional use of the hybrid OR was encountered in several hospitals, we calculated the utilization rate only based on the procedures that were performed with the fixed C-arm, ie, the hybrid OR was used as such and not as a conventional OR. The utilization rate was obtained by dividing the annual number of procedures with the fixed C-arm by the total annual procedures in the hybrid OR, multiplied with the utilization rate of a conventional OR.The average yearly costs for both ORs were divided by the average occupied hours per year. The sum of these costs and the average hourly personnel costs resulted in a cost per hour for both ORs. Those costs were converted to costs per minute (Supplementary file 4).

 To evaluate the cost drivers for the conventional and hybrid OR, a deterministic sensitivity analysis was performed. The main cost parameters were varied using the minimum and maximum values identified in the five hospitals. To evaluate the influence of personnel costs and overhead percentage, we used a 10% upper and lower limit.

 In addition, as a second sensitivity analysis, the utilization rate was varied from 30% to 100% for both ORs to show the influence on the costs per minute. As a final sensitivity analysis for the hybrid OR, the influence of different utilization rates of the fixed C-arm, varying from 10% to 100%, on the costs per minute were evaluated. In this analysis, it was assumed that the hybrid OR was optimally used (utilization rate of 92%) since the hybrid OR can also be used for surgeries without the use of a fixed C-arm (cross functional).

## Results

###  Base Case Cost-Analysis

Table 2 shows the costs per cost category, the total annual costs and costs per minute for both ORs. The average surface of a conventional and hybrid OR was 48.52 m^2^ and 85.36 m^2^, respectively.

**Table 2:** Base Case Results From the Bottom-Up Cost Analysis Including VAT and Presented in 2018 Euros

**Parameter**	**Conventional OR(Range)**		**Hybrid OR (Range)**	
Construction				
Costs per m^2^ OR	€12 804	(€10 064-€14 768)	€12 804	(€10 064-€14 768)
Total construction costs	€621 231	(€408 598-€841 776)	€1 092 915	(€526 347-€1 568 362)
Total annual costs	€71 673	(€47 141-€97 118)	€126 092	(€60 726-€180 945)
Inventory				
General inventory	€230 421^a^	(€190 027-€255 000)	€244 421^a^	(€190 027-€296 500)
Anesthesia	€128 308	(€65 000-€177 052)	€128 308	(€65 000-€177 052)
OR lights	€38 592	(€20 030-€73 134)	€42 598	(€32 000-€73 134)
Arm pendants	€69 262	(€40 000-€96 450)	€94 789	(€56 000-€135 333
OR table	€105 891	(€50 000-€155 160)	€265 222	(€215 000-€307 131)
Image routing system	€76 812	(€0-€122 059)	€93 126	(€0-€150 000)
X-ray radiation protection aprons	-	-	€18 384^a^	(€13 354-€23 682)
Fixed C-arm	-	-	€1 361 554	(€1 125 649-€1 550 000)
Total inventory costs	€649 375	(€365 057-€878 855)	€2 248 402	(€1 697 029-€2 712 832)
Total annual costs	€113 330	(€63 710-€153 379)	€433 241	(€329 938-€519 947)
Personnel				
Staff occupation per OR	Surgeon (1x)	€ 1.99	Surgeon (1x)	€ 1.99
	Anesthetist (0.5x)	€ 0.99	Anesthetist (0.5x)	€ 0.99
	Medical assistant (1x)	€ 0.62	Medical assistant (1x)	€ 0.62
	OR assistant (3x)	€ 1.87	OR assistant (3x)	€ 1.87
	Technician (0x)	€ 0	Technician (1x)	€ 0.62
Total personnel costs	€5.48	(€5.40-€5.56)	€6.10	(€6.01-€6.19)
Overhead				
Personnel costs (per minute)	€2.08	(€2.05-€2.11)	€2.32	(€2.28-€2.35)
Construction costs (annual)	€27 236	(€17 914-€36 905)	€47 915	(€23 076-€68 759)
Construction costs (per minute)	€0.24	(€0.16-€0.33)	€0.90	(€0.44-€1.30)
Total overhead costs	€2.32	(€2.21-2.41)	€3.22	(€2.72-€3.65)
Costs per minute (%) (range)				
Construction costs	€0.64 (6.8%)	(€0.42-0.87)	€2.38 (12.0%)	(€1.15-€3.42)
Inventory costs	€1.01 (10.7%)	(€0.57-€1.37)	€8.18 (41.1%)	(€6.23-€9.81)
Personnel costs	€5.48 (57.9%)	(€5.40- €5.56)	€6.10 (32.4%)	(€6.01-€6.19)
Overhead costs	€2.32 (24.6%)	(€2.21-2.41)	€3.22 (16.2%)	(€2.72-€3.65)
Total	€9.45	(€8.60-€10.23)	€19.88	(€16.10-€23.07)

Abbreviations: OR, operating room; VAT, value-added tax. ^a^based on only four hospitals. One hospital had in both conventional and hybrid OR no imaging routing system, therefore the minimal costs of the subcategory “image routing system” are zero.

 For the conventional OR the estimated annual construction costs were €71.673 (range €47.141-€97.118), the annual inventory costs were €113.330 (€63.710-€153.379) and the hourly personnel costs were €328 (€324-€333). Over construction costs, the annual overhead was €27.236 (€17.914-€36.905) and over personnel, the hourly overhead costs were €125 (€123-€127).

**Figure 1:**
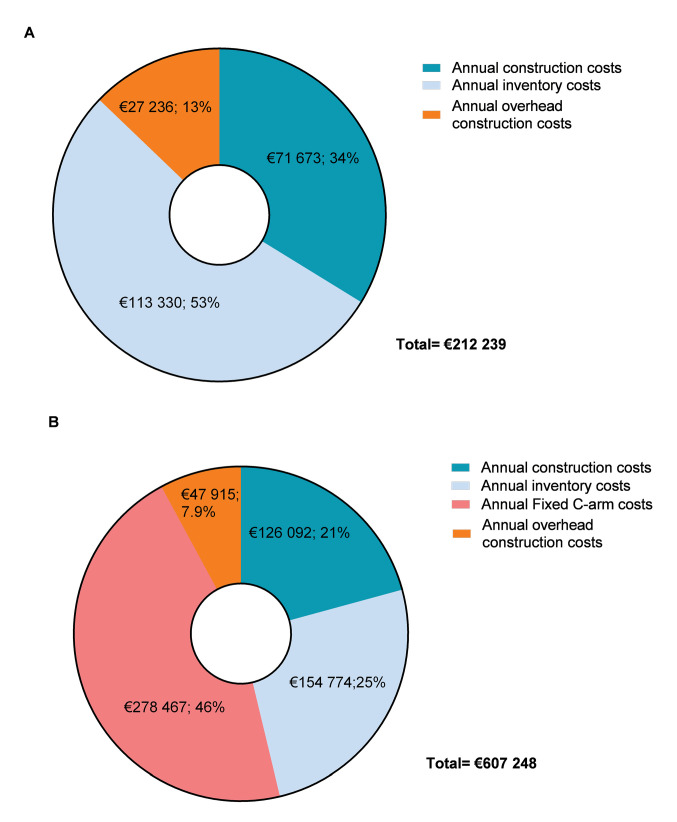
Total Fixed Costs of the Conventional OR and Hybrid OR. Abbreviation: OR, operating room.

For the hybrid OR the estimated annual construction costs were €126.092 (€60.726-€180 945), the annual inventory costs were €433.241 (€329.938-€519.947) and the hourly personnel costs were €366 (€361-€371). The overhead costs over the construction costs were €47.915 (€23.076-€68.759) and €139 (137-€141) over personnel per hour. The fixed C-arm accounted for 40% of the total fixed costs. Figure 1 shows the fixed annual costs related to both ORs.

**Figure 2:**
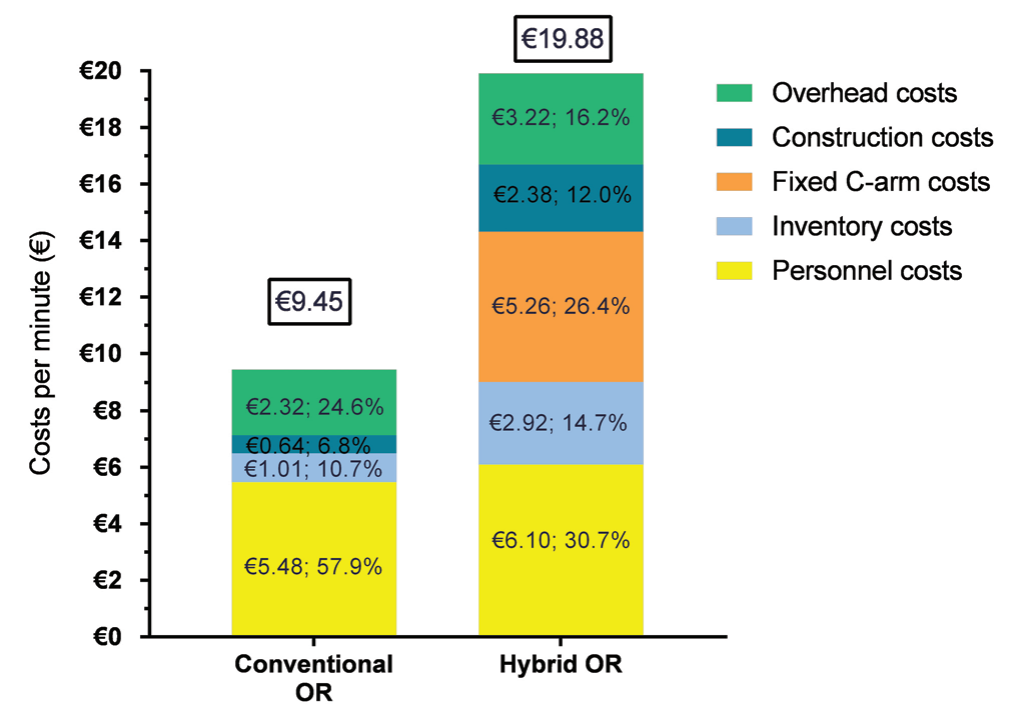
Total Costs Per Minute for the Conventional OR and Hybrid OR. In this figure the costs for the fixed C-arm are separated from the inventory costs. Abbreviation: OR, operating room.

The mean utilization rate for the conventional and hybrid OR were 92% (range 87%-96%) and 48% (range 14%-55%), respectively. These mean utilization rates were based on four hospitals because of a registration problem in one hospital. The detailed costs per cost category can be found in Table 2. For the conventional OR we found a total cost per minute of €9.45 (€8.60-€10.23), for which the personnel costs amounted to 58%. The total costs per minute for the hybrid OR were €19.88 (€16.10-€23.07). Since the costs for construction (12%) and inventory (41%) substantially increased compared to the conventional OR, the personnel costs amounted only to 31%. The costs per minute for both ORs are shown in Figure 2.

###  Cost Drivers

**Figure 3:**
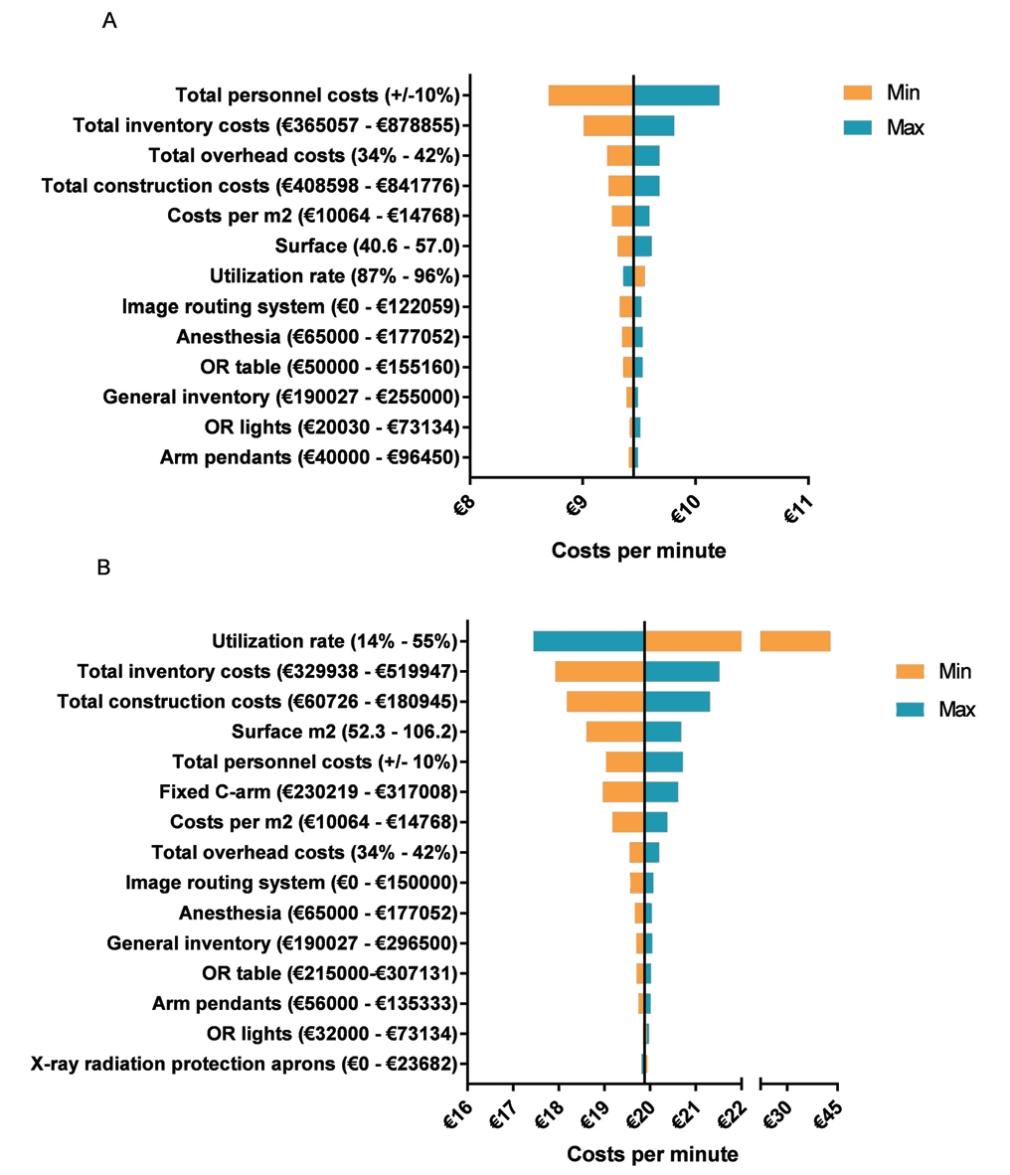
Results of the Deterministic Sensitivity Analysis for Both the Conventional OR (A) and Hybrid OR (B) in a Tornado Diagram. The values between brackets show the minimal and maximum values used in the sensitivity analysis. Abbreviation: OR, operating room.

**Figure 4:**
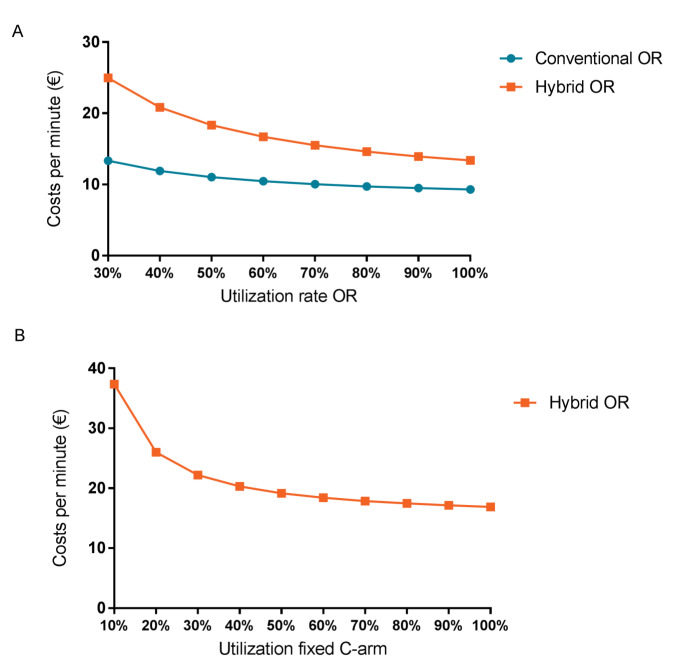
(A) Costs per minute of both conventional OR and hybrid OR by the utilization rate of the OR. It was assumed that the C-arm was used during all procedures performed in the hybrid OR. (B) Costs per minute of the hybrid OR by utilization rate of the fixed C-arm assuming an utilization rate of 92% for the hybrid OR. Abbreviation: OR, operating room.

Figure 3 shows the results from the deterministic sensitivity analysis. The total personnel costs had most influence on the total costs of the conventional OR, followed by total inventory and overhead costs. The inventory costs were mainly driven by the costs for imaging routing system, anesthesia and OR table costs. For the hybrid OR, the utilization rate had most influence on the total costs, followed by the inventory costs and construction costs. The inventory costs were mainly driven by the costs of the fixed C-arm. Figure 4a shows that less efficient utilization of the conventional OR, for instance, 50%, results in an increase of €1.73 per minute compared to a fully used OR (100%). For the hybrid OR a difference in cost per minute of €4.96 was seen, comparing a utilization rate of 50% to 100%. Figure 4b shows that using the fixed C-arm only half of the time, assuming an utilization rate of 92% for the hybrid OR, results in a total OR costs of €19.17 per minute.

###  Calculation Model

As seen by Figure 3, the input parameters such as specific inventory costs can have a large influence on our calculated costs per minute. As those costs are different per hospital and country, a calculation model (Supplementary file 1) was attached to this article in which input parameters can be adjusted.

## Discussion

 The identified costs per minute for the conventional OR were €9.45 (€8.60-€10.23) and for the hybrid OR €19.88 (€16.10-€23.07), measured in a setting of five hospitals in the Netherlands. The difference between the two ORs (€10.43) is mainly explained by higher inventory costs for the hybrid OR namely, €7.17. Noteworthy is that although advanced imaging technology is expensive, personnel costs remain an important element in the costs of both ORs (58% and 31% of the costs for the conventional and hybrid OR, respectively). For the hybrid OR, the construction costs became an important element compared to the conventional OR (increase from 7% to 12% for the per-minute costs), which is explained by the larger room needed.

Comparing our results with available results in the literature is challenging because existing studies include different input parameters and adopt different perspectives. For instance, a mean cost of $37.45 per minute was found for a conventional OR in the United States.^13^ This study incorporated costs for disposables and had a top-down perspective which presumably explains the difference to our results. Raft et al found a cost per minute of €10.80 for using the OR and post-anesthesia care unit by a top-down cost analysis. Since they also incorporated the costs of the post-anesthesia care unit, medicines, and disposables used during operations the results are hard to compare.^14^ As purchasing power parity differences are rather modest, this might reflect differences in norms for capital expenditure between US and European hospitals; it is however likely that the relative difference between conventional and hybrid ORs is comparable.

To date, no studies that solely evaluated the costs of using a hybrid OR were identified. However, some studies compared a specific hybrid surgery with a non-hybrid surgery in which intervention costs were taken into account.^8,26-28^ Two studies showed that the hybrid approach reduced operating time, length of stay and resulted in less resource use for cardiovascular surgery. They however neglected the potential higher intervention cost for using the hybrid OR.^8,27^ Another cardiovascular study evaluated the additional costs for the hybrid approach and reported higher costs, but took a reduction of procedure time for the hybrid approach into account in reporting the total costs. Therefore, it is not possible to deduce the additional costs only for the use of the hybrid OR.^26^

To the best of our knowledge, this is the first bottom-up cost analysis that provides insight into the costs and cost drivers for both the conventional and the hybrid OR. The main strength of our study is that we evaluated the costs transparently by (1) performing a bottom-up cost-analysis, (2) specifically stating results for the different cost categories, (3) comprehensively describing the methods and calculations used in the analysis, and (4) enclosing a calculation model in which input parameters can be changed to specific settings for instance, to incorporate the costs of a mobile C-arm in the conventional OR or include costs for endoscopic devices and disposables (Supplementary file 1). Another strength in this study was the comparison and anonymous discussion of the data of the five hospitals, academic and non-academic, to result in a general cost price for the Dutch setting.

The present study has several limitations. First, the base case analysis assumed that personnel had no idle time and a minimal team needed for running the (hybrid) OR was taken into account which underestimates the actual costs. Furthermore, the correction for irregular hours on personnel costs was assumed equal for the conventional and hybrid OR. This might underestimate the personnel costs for the hybrid OR, when this OR is often used during irregular hours (eg, in trauma interventions).This underestimation could influence our results as personnel costs are an important factor driving the costs of both ORs (Figure 3). This topic should therefore be evaluated in the future. Second, the construction costs were evaluated based on square meters, to avoid hospital differences and choices in design. Especially for the hybrid OR, these may not reflect the actual costs since we did not evaluate the specific costs of the conditional adaptations, eg, a larger room, lead lining in the walls and additional installations.^29^ In addition, the size of the hybrid OR differed substantially in our analysis (range: 52.3-106.2) by using the average surface we may underestimate these costs as well. Third, as we only included Dutch hospitals, the generalizability of our results may be limited. Therefore, we contacted one additional hospital in a high-income country (Oslo University Hospital), which recently built innovative operating suites, to verify the construction and inventory costs. The costs of this hospital seemed comparable to the Dutch setting. Also, their construction costs, which were based on the actual construction costs, were in line with our estimates. Finally, overhead costs are very hard to obtain using a bottom-up methodology, therefore we chose a fixed percentage over the construction costs and personnel costs defined in the Dutch manual for cost calculations.^20^ This could result in an over or underestimation of the indirect costs. However, ranging the overhead over 34% and 42% (base case 38%) had a relatively minor influence on the total costs per minute. Also, the maintenance costs for imaging equipment were difficult to obtain, especially when considering that additional staff is necessary to support the maintenance activities within the hospitals. The estimated maintenance of 8%, which was obtained through expert consultation, might therefore be an underestimation. As in our sensitivity analysis the upper and lower values of the C-arm showed a relatively limited effect on the results, we expect that a different percentage for maintenance costs would not change our conclusions.

The following example gives an impression on the added value per patient needed to consider the use of the fixed C-arm (in the hybrid OR) cost-effective. From the estimated costs for the conventional and hybrid OR, we can calculate the base case incremental costs for the hybrid OR per minute (€10.43). Assuming a surgical procedure of approximately 2.5 hours, for example, an endovascular aneurysm repair, results in an incremental cost of €1.565 per procedure. These incremental costs only incorporated the additional costs of using the hybrid OR, not additional materials that might be needed. When a cost-effectiveness threshold of €50.000 per quality-adjusted life year is assumed,^30^ a minimum gain of 0.031 quality-adjusted life year is required. This means, using this simplified calculation, that for performing an endovascular aneurysm repair in the hybrid OR, at least 11 days in perfect health should be gained to be considered cost-effective compared to a conventional OR. This is based on the utilization rate of 43%. When calculating the incremental costs using a different procedure duration, an estimation can be made for other procedures as well. Based on this calculation it may seem difficult for the hybrid OR to become cost-effective, however by increasing the utilization rate or comparing the intervention to a different technique, eg, laparoscopy which results in lower incremental costs, it becomes more likely that the hybrid OR is cost-effective. “This suggestion is strengthened by a recent evaluation of a navigation system that is used during surgery, which requires the hybrid OR. In removing locally recurrent rectal tumors the use of a navigation system in the hybrid OR has the potential to become cost-effective. However, the level of cost-effectiveness of the navigation system is also strongly dependent on the utilization rate of both the navigation system and the hybrid OR.”^30^

## Conclusion

This study identified that the main factors influencing the OR costs are: total inventory costs, total construction costs, utilization rate, and total personnel costs. Therefore, our results could inform surgeons, decision and policy-makers in hospitals on the adoption and optimal utilization of new (hybrid) ORs. Although it seems that hospitals have to strive to use the fixed C-arm as often as possible, one should keep in mind that using the hybrid OR should add value to the patient. To evaluate the added value, the calculation model (Supplementary file 1) can be used to evaluate the costs and estimate the required added value for a specific setting and/or country. These estimations can be useful in identifying the most promising procedures performed in the hybrid OR to steer future research directions.

###  Future Directions

As it is expected that the number of hybrid ORs will rise worldwide^1^, and those are likely to become more advanced (eg, including a magnetic resonance imaging) it is important to evaluate its cost-effectiveness. Therefore, in the near future prospective comparative studies have to be performed to evaluate the actual benefits of using these advanced ORs in terms of complication rates, efficiency, and survival. Those outcomes can be used to identify interventions that yield the most from the advances of the hybrid OR, as well as informing cost-effective usage of the hybrid OR in general. Finally, in order to assess the generalizability of our results to non-European or low- and middle-income countries, it would be valuable to validate our calculation model from a different perspective (eg, US perspective).

## Acknowledgements

 The authors would like to thank the all participating hospitals that contributed to this study by delivering their hospital data.

## Ethical issues

 Ethical approval was not required for this study, as no human participants were involved.

## Competing interests

 All authors report no conflicts of interest. MR, JPCG, and SP have received an unrestricted research grant from Siemens Healthcare GmbH for other but related research projects.

## Authors’ contributions

 Conception and design: JPCG, VR, SP, and ML. Acquisition of data: SP and ML. Analysis and interpretation of data: SP, ML, JPCG, VR, MR, and WHvH. Drafting of the manuscript: SP and ML. Critical revision of the manuscript for important intellectual content: SP, ML, JPCG, VR, MR, WHvH, TR, and LP. Statistical analysis: SP and ML. Supervision: JPCG, VR, MR, and WHvH.

## Authors’ affiliations

^1^Department of Operating Rooms, Radboud Institute for Health Sciences, Radboud University Medical Center, Nijmegen, The Netherlands. ^2^Division of Psychosocial Research and Epidemiology, The Netherlands Cancer Institute-Antoni van Leeuwenhoek, Amsterdam, The Netherlands. ^3^Department of Health Technology and Services Research, University of Twente, Enschede, The Netherlands. ^4^Department for Health Evidence, Radboud Institute for Health Sciences, Radboud University Medical Center, Nijmegen, The Netherlands. ^5^Department of Surgery, The Netherlands Cancer Institute-Antoni van Leeuwenhoek Hospital, Amsterdam, The Netherlands. ^6^Department of Medical Technology, Isala Hospital, Zwolle, The Netherlands.

## Supplementary files


Supplementary file 1. Calculation Model.

Supplementary file 2. Characteristics of Participating Hospitals.

Supplementary file 3. Example of the Calculation of the Construction Costs.

Supplementary file 4. Formulas to Calculate the Costs Per OR.
