# Supplementary material for: Understanding the Costs of Surgery: A Bottom-Up Cost Analysis of Both a Hybrid Operating Room and Conventional Operating Room
Source: Int J Health Policy Manag. 2020 Jul 27;11(3):299–307. doi: 10.34172/ijhpm.2020.119 (PMC9278478; doi:10.34172/ijhpm.2020.119)
Supplement: Supplementary file 2 — Characteristics of Participating Hospitals. [file ijhpm-11-299-s002.pdf]

Supplementary 2. Characteristics of Participating Hospitals

Hospital	Number of beds	Number of ORs	Involved experts
A	212	12	Anesthetist<br>Department building management<br>Financial department<br>Head of the OR<br>Staff involved in capacity measurements
B	766	22	Head of the OR<br>Department building management<br>Board of directors<br>Staff involved in capacity measurements
C	593 (2018)	20	Head of the OR<br>Coordinator OR technology<br>Managing director OR department<br>Department building management<br>Financial department<br>Staff involved in capacity measurements
D	776 (n.d.)	14	Clinical physicist<br>Medical technician<br>Staff involved in capacity measurements<br>Project manager OK
E	630 (2017)	16	Clinical physicist<br>Staff involved in capacity measurements<br>Technical coordinator<br>Coordinator surgery in the OR department
