# Supplementary material for: Understanding the Costs of Surgery: A Bottom-Up Cost Analysis of Both a Hybrid Operating Room and Conventional Operating Room
Source: Int J Health Policy Manag. 2020 Jul 27;11(3):299–307. doi: 10.34172/ijhpm.2020.119 (PMC9278478; doi:10.34172/ijhpm.2020.119)
Supplement: Supplementary file 3 — Example of the Calculation of the Construction Costs. [file ijhpm-11-299-s003.pdf]

Supplementary 3. Example of the calculation of the construction costs

The following formulas were mentioned in the article:

1 $$\sum C_{M2\ not\ labeled\ as\ OR} = \sum M^2_{functionality\ i} * C_{differentiated\ i}$$

2 $$\frac{(\sum M^2_{OR\ department} * C_{M2\ costs\ OR\ department}) - \sum C_{M2\ not\ labeled\ as\ OR}}{\sum M^2_{OR\ within\ OR\ department}}$$

This supplement gives an example of the construction cost calculation. The "C" in the formula refers to costs and the "M^2"to square meters.

Picture 1 shows a simplified OR department containing corridors, ORs, a storage room, offices, the holding, the recovery and a place to have lunch and coffee.

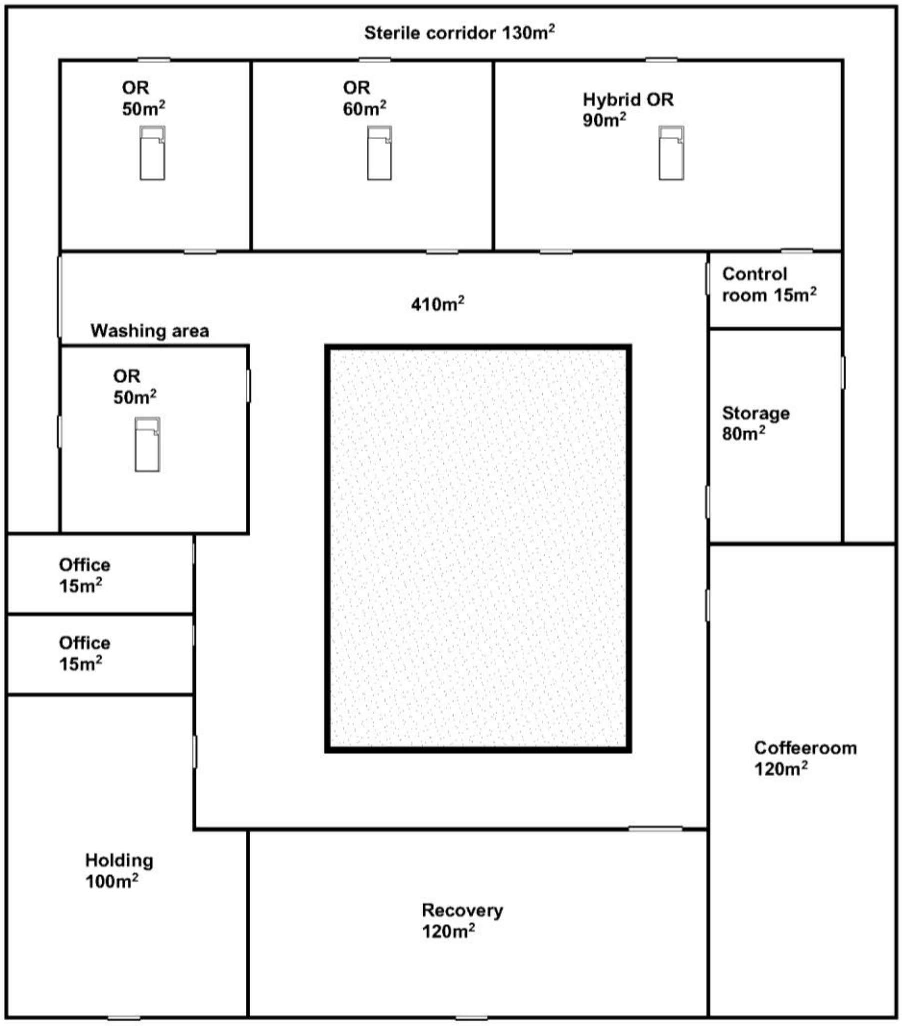

Picture 1

The total surface of this OR department is 1195m2.

Costs for constructing a m2 = €3479
Costs for constructing a m2 in an OR department = €5566 (differentiated with 160%)

The first part of the formula focuses on the surface of the OR department that is not belonging to the OR.
In this example that means: 30m2 office, 100m2 holding, 120m2 recovery, 120 m2 coffee room, 80m2 storage room, 410m2 standard corridor which sums up to: 860m2
The sterile corridor and control room were included in the surface belonging to the OR = 395m$^{2.}$

1 $\sum C_{M2\ not\ labeled\ as\ OR} = \sum M^2_{functionality\ i} * C_{differentiated\ i}$
Filling in this formula gives the table below:

$M^2_{functionality\ i}$	**Differentiated with**	$(C_{differentiated\ i}) * M^2_{functionality\ i}$
Moffice = 30m2	100%	= (3479 * 100%) * 30 = 104.370
Mholding = 100m2	140%	= (3479 * 140%) * 100 = 487.060
Mrecovery = 120m2	140%	= (3479 * 140%) * 120 = 584.472
Mcoffee room = 120m2	75%	= (3479 * 75%) * 120 = 313.110
Mstorage = 80m2	75%	= (3479 * 75%) * 80 = 208.740
Mcorridor = 410m2	75%	= (3479 * 75%) * 410 = 1.069.792

	$\sum C_{M2\ not\ labeled\ as\ OR}$	= € 2.767.544

This number is used in the next formula

2 $$\frac{(\sum M^2_{OR\ department} * C_{M2\ costs\ OR\ department}) - \sum C_{M2\ not\ labeled\ as\ OR}}{\sum M^2_{OR\ within\ OR\ department}}$$

Two of the four variables were given at the beginning:
M^2 OR department = 1195m^2
M^2 costs of OR department =€5566

The final variable can be extracted from Picture 1:
m^2 of OR within OR department = + 50 + 60 + 90 + 15 + 130 = 395 m^2

We can fill in the formula:
= ((1195*5566) – 2.767.544) / 395
= € 9832

In this example constructing a m^2 of an OR would cost €9832.
