# Supplementary material for: Understanding the Costs of Surgery: A Bottom-Up Cost Analysis of Both a Hybrid Operating Room and Conventional Operating Room
Source: Int J Health Policy Manag. 2020 Jul 27;11(3):299–307. doi: 10.34172/ijhpm.2020.119 (PMC9278478; doi:10.34172/ijhpm.2020.119)
Supplement: Supplementary file 4 — Formulas to Calculate the Costs Per OR. [file ijhpm-11-299-s004.pdf]

Supplementary 4. Formulas to calculate the costs per OR

In this analysis the formulas used to calculate the OR costs for both the hybrid and conventional OR were as follows:

Costs of the OR per hour =

$$\frac{Annual\ construction\ costs + annual\ inventory\ costs + annual\ overhead\ costs}{Utilization\ rate * available\ production\ hours\ of\ OR}$$

$$+hourly\ personnel\ costs$$

Costs of the OR per minute =

$$\frac{Costs\ of\ the\ OR\ per\ hour}{60}$$
